# Research trends in the application of artificial intelligence in nursing of chronic disease: a bibliometric and network visualization study

**DOI:** 10.3389/fdgth.2025.1608266

**Published:** 2025-06-18

**Authors:** Chao Du, Jing Zhou, Yuexin Yu

**Affiliations:** Department of Reproductive Medicine, General Hospital of Northern Theater Command, Shenyang, China

**Keywords:** artificial intelligence, machine learning, chronic disease, nursing, cancer

## Abstract

**Purpose:**

The incidence of chronic diseases is increasing annually and exhibits a trend of multimorbidity, posing significant challenges to global healthcare and nursing. The rapid rise of artificial intelligence has provided broad application prospects in the field of chronic disease care. However, with the increasing number of related studies, there is a lack of systematic review and prediction of future trends in this area. Bibliometric methods provide possibility for addressing this gap. This study aimed to investigate the current status, hot topics, and future prospects of artificial intelligence in the field of chronic disease care.

**Methods:**

Literature related to artificial intelligence and chronic disease care was retrieved from the Web of Science Core Collection database, published between 2001 and 31 December 2023. Bibliometric analysis and visualization was conducted using CiteSpace 5.7.R5 and VOSviewer 1.6.19 to analyze countries/regions, institutions, journals, references, and keywords.

**Results:**

A total of 2438 articles were retrieved, indicating an explosive growth in publications over the past five years. The United States emerged as the earliest adopter of research in this domain (since 2002) and contributed the most publications (490 articles), with IEEE ACCESS being the most cited journal. Hot application areas of artificial intelligence in chronic disease care included “diabetic retinopathy”, “heart disease prediction”, “breast cancer”, and “skin cancer”. Major research methodologies encompassed “machine learning”, “deep learning”, “neural network”, and “text mining”. Potential future research hotspots include “internet of medical things”.

**Conclusion:**

This study unveils the current status and development trends of artificial intelligence in chronic disease care, offering novel insights for future artificial intelligence application research.

## Introduction

Chronic diseases, abbreviated as Chronic Non-communicable Diseases (CNCDs), refer to a category of long-term, incurable conditions that typically do not resolve on their own and are rarely completely cured. Common CNCDs include cancer, cardiovascular diseases, diabetes, and chronic respiratory diseases (1). Chronic diseases are the leading causes of disability and death, accounting for 74% of global deaths (equivalent to 41 million deaths annually). It is projected that by 2030, this figure will rise to 52 million. Moreover, chronic diseases consume 90% of annual healthcare expenditures, and by 2030, the cumulative global economic losses are estimated to reach $47 trillion (2, 3). Therefore, chronic diseases are increasingly receiving attention worldwide, posing significant burdens on individuals, families, governments, and healthcare systems. The characteristics of chronic diseases include frequent multimorbidity, requiring long-term symptom management (often lifelong) and continuous care. Studies have indicated that current chronic disease care lacks continuity in individualized care and explanations of patients' illnesses and treatments (4), thereby increasing the difficulty of chronic disease management. Over the past decade, artificial intelligence has experienced rapid development and has been widely adopted in the healthcare industry. This offers new options for chronic disease care. Multiple studies have suggested that the emergence of artificial intelligence technology not only holds promise for improving the quality of patient care and health outcomes by reducing human errors but also has the potential to free up time for clinicians and healthcare workers from routine and repetitive tasks, enabling them to focus on more complex duties (5, 6).

Artificial Intelligence (AI) is a new scientific technology that encompasses the theory, methods, techniques, and application systems developed for simulating, extending, and expanding human intelligence (7). AI comprises multiple branches, such as machine learning (ML) (8) and natural language processing (NLP) (9). ML, one of these branches, includes various algorithms categorized broadly into supervised learning, unsupervised learning, and reinforcement learning. Supervised learning utilizes labeled datasets to train algorithms for data classification or accurate result prediction. Unsupervised learning involves using unlabeled data to learn about the distribution of data or relationships between data, with main tasks including dimensionality reduction and clustering. Reinforcement learning entails programs or agents learning a mapping from environment to action through continuous interaction with the environment, aiming to maximize cumulative rewards. NLP, another distinct branch of artificial intelligence, utilizes computational language techniques to analyze vast amounts of natural language at specific language structure levels, including text and speech. With the proliferation of electronic health records and the advent of the era of health big data, NLP has been applied across various aspects of nursing care. Despite the increasing research on the application of AI in chronic disease care, scholars have conducted reviews on the current research status (10, 11), but there remains a lack of elucidation on the research structure in this field and predictions of future development trends.

Bibliometrics is a quantitative statistical analysis method for studying literature. Through this method, we can quickly understand the development process, collaborative relationships, research hotspots, and predict future development trends in a particular field. This method has been applied in various fields (12–14). In this study, we conducted a systematic analysis of recent literature on AI and CNCDs using Citespace software developed by Chaomei Chen's team (15) and VOSviewer software developed by Van Eck et al. (16). This analysis aims to enable researchers to better grasp the current research status and overall trends in this field, providing a reference for subsequent research endeavors.

## Material and methods

### Data source and search

The literature reviewed in this study was retrieved from the Web of Science Core Collection (WoSCC) database on February 27, 2024. The literature was searched using the following criteria: (TS = “Nursing” OR “Health care”) AND (TS = “Artificial Intelligence” OR “machine learning” OR “deep learning” OR “natural language processing” OR “robot” OR “Neural Networks” OR “Intelligent Tutoring” OR “Intelligent Agents” OR “Voice Recognition” OR “Text Mining”) AND (TS = “chronic disease” OR “cancer” OR “heart disease” OR “diabetes” OR “hypertension” OR “chronic obstructive pulmonary disease”). The search criteria were limited as follows:

(1) Document type: articles;
(2) No species restriction;
(3) Language: English
(4) Publication date: 2001–2023

For articles meeting these inclusion criteria, all records including titles, authors, abstracts, keywords, and references were exported and saved as a plain text file named “download_txt”. Subsequently, these files were imported into CiteSpace 5.7.R5 for further analysis.

### Data analysis and visualization

We conducted bibliometric analysis and visualization using CiteSpace 5.7.R5, developed by Professor Chaomei Chen. CiteSpace facilitates data visualization through features such as co-citation networks, co-word networks, and author co-citation analysis, presenting the structure, patterns, and distribution of scientific knowledge in a particular discipline or field.

Specific parameters for CiteSpace 5.7.R5 software were set as follows: Time slicing: Jan 2002 to Dec 2023, Years per clice:1. Term source: Title, Abstract, Author Keywords and Keyword Plus, Node type: Author, Institution, Country, Keyword, Reference, Cited Author and Cited Journal, Link strength: Cosine, Selection Criteria: g-index, k = 25. Pruning: Pathfinder, Pruning the merged network. Visualization: Cluster View-Statics, Show Merged Network.

Additionally, VOSviewer 1.6.19 was used for keyword clustering analysis, time visualization, and research density visualization analysis, with parameters set to default settings. In the clustering analysis, node color represents the category to which it belongs. Keyword co-occurrence network analysis was utilized to examine the structure and distribution of research hotspots, while the timeline of keyword occurrence and research density were used to delineate the development trajectory of the field and predict future research trends.

## Results

### Results of annual publication analysis

From WoSCC, a total of 2438 articles were exported. No relevant articles were found in 2001. The annual publication output increased from 2 articles in 2002 to 737 articles in 2023, with citation counts rising from 2 to 14979 over the same period. There was a surge in publications since 2018, indicating widespread attention to research in the field of AI applied to chronic disease care over the past five years. Upon analyzing the fields in which the articles were categorized, it was found that the Computer Science Information System domain had the highest publication output, with 384 articles, followed by Engineering Electrical Electronic (307 articles), Medical Informatics (303 articles), Computer Science Interdisciplinary Applications (238 articles), and Health Care Sciences Services (219 articles).

Data were preprocessed using CiteSpace 5.7.R5, resulting in the exclusion of 1 Biographical-Item, 35 Editorial Material, 2 Letter, 8 Meeting Abstract, and 501 Review articles, leaving a final inclusion of 1968 articles categorized as Articles. Statistical analysis revealed that multiple authors had the highest publication output, including SHAHADAT UDDIN, MUHAMMAD ADNAN KHAN, and ABDULLAH MOHAMED, each with 6 articles. The United States emerged as the top country/region in terms of publication output, with 490 articles, while Harvard Medical School was the leading institution with 40 articles. The top 5 countries/regions, institutions, and authors by publication volume are presented in Table 1.

**Table 1:** Top 5 authors, institutions and countries/regions, by number of publications.

Ranking	Author	Number of publications	Centrality	Year
1	SHAHADAT UDDIN	6	<0.01	2021
2	MUHAMMAD ADNAN KHAN	6	<0.01	2021
3	ABDULLAH MOHAMED	6	<0.01	2022
4	RADWA MARZOUK	6	<0.01	2022
5	JOSEPH H SCHWAB	6	<0.01	2018
Ranking	Institution	Number of Publications	Centrality	Year
1	Harvard Med Sch	40	0.04	2016
2	King Saud Univ	36	<0.01	2019
3	King Abdulaziz Univ	26	<0.01	2029
4	Massachusetts Gen Hosp	25	0.17	2014
5	Vellore Inst Technol	23	0.04	2022
Ranking	Country/Region	Number of Publications	Centrality	Year
1	USA	490	0.30	2002
2	PEOPLES R CHINA	231	0.16	2012
3	INDIA	229	0.19	2015
4	SAUDI ARABIA	179	0.13	2017
5	ENGLAND	117	0.50	2006

### Results of co-cited journal analysis

We conducted co-citation analysis on all journals. In the co-citation network graph of journals, each node represents a journal, with the size of the node indicating the frequency of citations. When two nodes are simultaneously cited by a third node, it indicates a co-citation relationship, which is represented by a connecting line, with the thickness of the line representing the frequency of co-citation between the two nodes.

**Figure 1:**
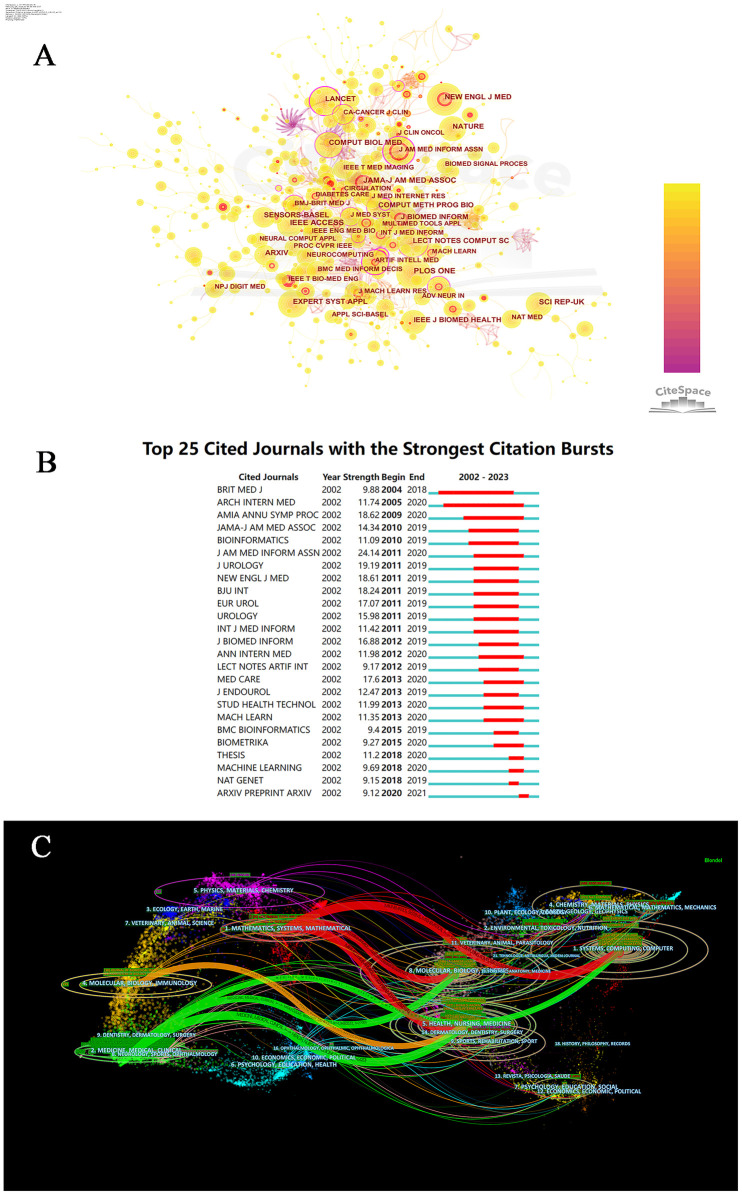
Analysis of Co-cited journals involved in artificial intelligence and nursing of chronic Non-communicable diseases. **(A)** The visualization map showing academic journals publishing research. **(B)** Top 25 cited journals with the strongest citation bursts. **(C)** A dual-map overlay of journals related to research on artificial intelligence and nursing of chronic non-communicable diseases.

The journal with the most citations is *IEEE ACCESS* (534 citations). Journals with more than 150 citations are detailed in Figure 1A. We observed that the most cited articles not only appeared in top-tier medical journals (such as *JAMA* and *New England Journal of Medicine*) but also in journals, series, or platforms related to computer science, such as *ARXIV* and *EXPERT SYST APPL*. This indicates that research related to AI and chronic disease care is not only diverse in terms of cited journals but also rapidly evolving, attracting significant attention from both medical and computer science fields.

Through the detection of journal bursts, we found that the earliest appearance of a burst citation journal was the *British Medical Journal* in 2004, which continued until 2018. Figure 1B illustrates the top 25 journals with the strongest burst citations, indicating the sustained rapid development of this field over the past two decades. Of note, the most recent occurrence of a burst node was *ARXIV*, a website that collects preprints of papers in physics, mathematics, computer science, biology, and quantitative finance, bypassing traditional peer-review processes and facilitating rapid sharing of research outcomes. This suggests that research in this field is closely aligned with developments in computer science and is exhibiting an increasingly rapid growth trend.

The dual-map overlay results of the journals illustrate the relative position of the research topic compared to the main disciplines. Each point on the map represents a journal, with the map divided into two parts: the left side represents citing journals, and the right side represents cited journals. The curves depict citation lines, providing a comprehensive view of citation patterns. In the left-side map, ellipses represent the number of publications corresponding to a journal and indicate the ratio of authors to publications; the length of the ellipse represents the number of authors, while the width represents the number of publications. The curves between the left and right parts of the map represent citation links, and the trajectories of these links offer insight into interdisciplinary relationships in the field. The z-scores function highlights stronger and smoother trajectories, with higher scores represented by thicker connecting lines.

From Figure 1C, it can be observed that publications in the Medicine/Medical/Clinical domain (green trajectory) are significantly influenced by publications from the Systems/Computing/Computer (z = 2.94, f = 1,862), Molecular/Biology/Genetics (z = 2.95, f = 2,700), and Health/Nursing/Medicine (z = 7.59, f = 6,420) domains. Additionally, publications in the Molecular/Biology/Immunology domain (orange trajectory) are influenced by publications from the Health/Nursing/Medicine (z = 1.95, f = 1,888) domain. Moreover, publications in the Mathematics/Systems/Mathematical domain (red trajectory) are influenced by publications from the Systems/Computing/Computer (z = 3.62, f = 3,242), Health/Nursing/Medicine (z = 4.23, f = 3,740), and Molecular/Biology/Genetics (z = 2.42, f = 2,270) domains.

### Analysis of co-cited references

**Figure 2:**
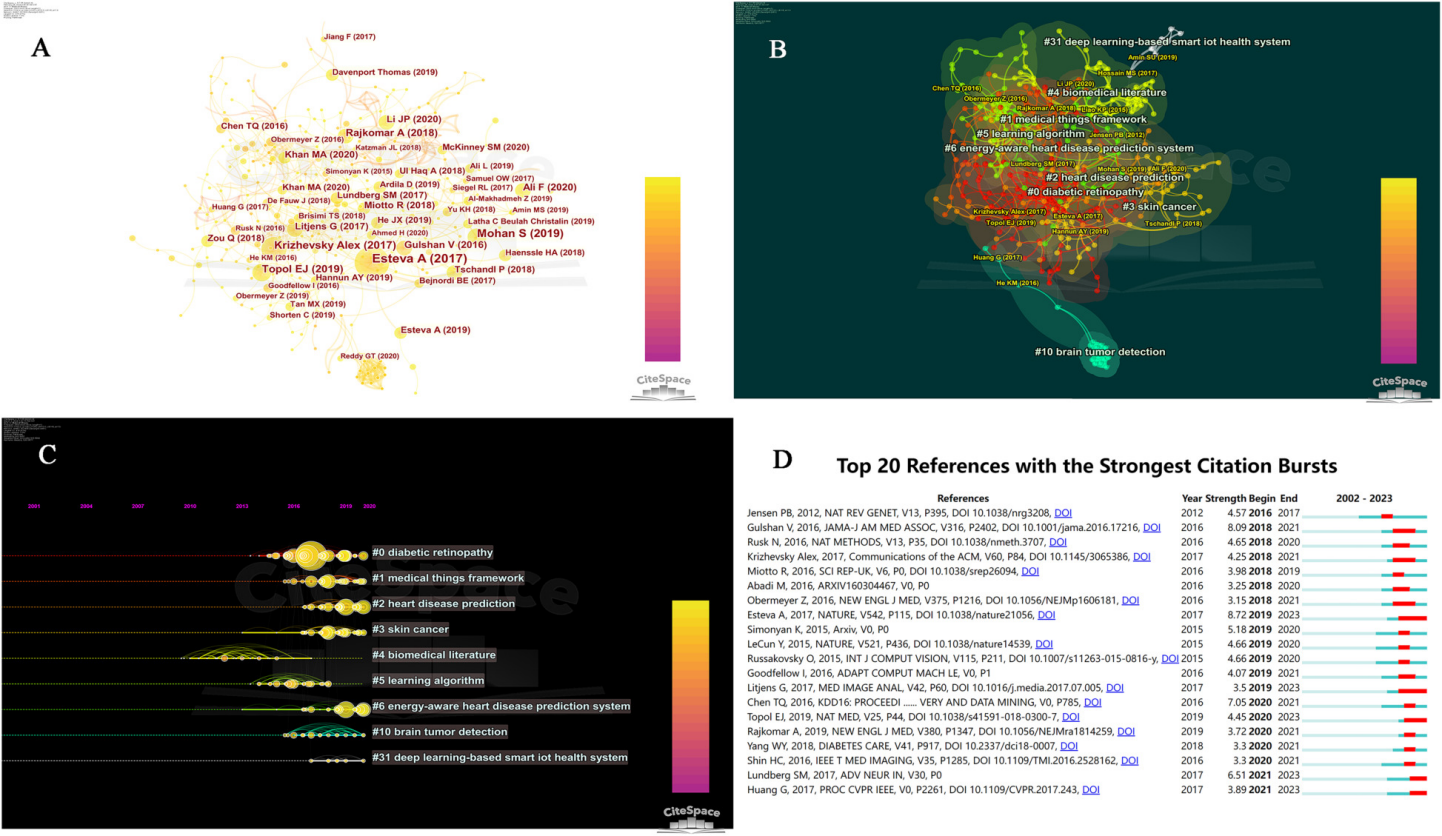
Citation analysis of AI and chronic disease nursing. **(A)** Co-citation network diagram of references related to AI and chronic disease nursing; **(B)** citation cluster analysis results elated to AI and chronic disease learning; **(C)** citation clustering fisheye diagram; **(D)** top 20 references with the strongest citation burst. Note: AI, artificial intelligence.

To gain further insight into the specific content of research on AI and chronic disease nursing, we conducted co-citation analysis using CiteSpace5.7.R5 on the references. The results, as shown in Figure 2A, depict each node representing a cited document, with node size indicating the number of citations. Different colors of concentric circles represent the citation years, where the width of the circles corresponds to the number of citations within that year. Node annotations display the authors and publication dates of the cited articles. The most cited article is by Esteva A et al. (17), titled “Dermatologist-level classification of skin cancer with deep neural networks” published in *Nature*. This study utilized deep neural networks for skin cancer diagnosis, achieving expert-level accuracy and garnering 73 citations. The top 10 most cited articles are listed in Table 2 (17–26). We conducted clustering analysis on articles cited more than 5 times, resulting in 9 clusters with a clustering index of Modularity Q = 0.95 and Weighted Means Silhouette = 0.97, indicating significant modularity and coherence. The clustering results are reasonable, with main themes including “diabetic retinopathy”, “medical things framework”, “heart disease prediction”, and “skin cancer”, as detailed in Figure 2B. The fisheye view visually displays the time nodes of each cluster, with most nodes appearing after 2010 (Figure 2C). Through burst detection of citations, we observed a higher frequency of burst citations after 2015, with Esteva A et al.'s study exhibiting the highest burst intensity of 8.72, highlighting its significant importance in the field. The most recent burst citation is from Huang G et al. (27), published in the *IEEE Conference on Computer Vision and Pattern Recognition* (CVPR) in 2017, warranting attention in future research, as detailed in Figure 2D.

**Table 2:** Top 10 co-cited references in the research of artificial intelligence and chronic disease nursing.

Ranking	Year	First author	Title	Journal	Citations
1	2017	Esteva A	Dermatologist-level classification of skin cancer with deep neural networks	*NATURE*	73
2	2019	Mohan S	Effective Heart Disease Prediction Using Hybrid Machine Learning Techniques	*IEEE ACCESS*	50
3	2017	Krizhevsky Alex	ImageNet classification with deep convolutional neural networks	*Communications of the ACM*	39
4	2019	Topol EJ	High-performance medicine: the convergence of human and artificial intelligence	*NAT MED*	36
5	2018	Rajkomar A	Scalable and accurate deep learning with electronic health records	*NPJ DIGIT MED*	30
6	2016	Gulshan V	Development and Validation of a Deep Learning Algorithm for Detection of Diabetic Retinopathy in Retinal Fundus Photographs	*JAMA-J AM MED ASSOC*	28
7	2020	Ali F	A smart healthcare monitoring system for heart disease prediction based on ensemble deep learning and feature fusion	*INFORM FUSION*	26
8	2020	Li JP	Heart Disease Identification Method Using Machine Learning Classification in E-Healthcare	*IEEE ACCESS*	24
9	2017	Litjens G	A survey on deep learning in medical image analysis	*MED IMAGE ANAL*	24
10	2018	Miotto R	Deep learning for healthcare: review, opportunities and challenges	*BRIEF BIOINFORM*	23

### Hotspots and frontiers analysis

**Figure 3:**
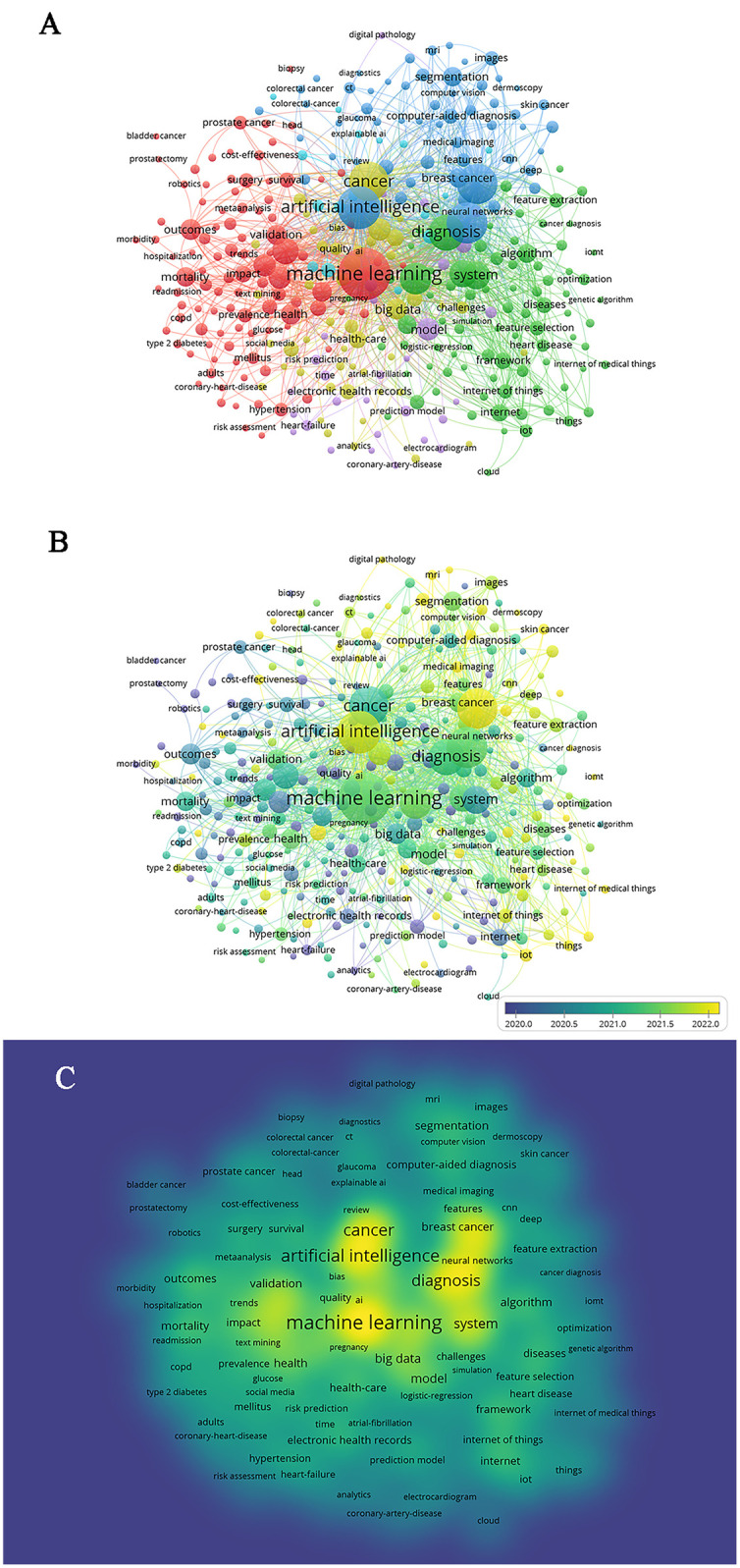
Co-occurrence network diagram of key words related to artificial intelligence and chronic disease nursing. **(A)** The cluster analysis of keywords related to artificial intelligence and chronic disease nursing. **(B)** Overlay visualization of keywords. **(C)** Density visualization of keywords.

In this study, VOSviewer was utilized to conduct co-occurrence clustering analysis of keywords, resulting in the classification of all keywords into 6 clusters. Cluster 1 includes terms such as “machine learning”, “health”, “outcomes”, and “mortality”, representing research on the application of machine learning in healthcare and prognosis prediction, depicted in red. Cluster 2 comprises terms like “artificial intelligence”, “breast cancer”, “computer aided diagnosis”, and “segmentation”, representing research in the fields of artificial intelligence and cancer-assisted diagnosis, depicted in blue. Cluster 3 includes terms like “diagnosis”, “system”, “algorithm”, and “feature extraction”, representing research on various algorithms in system diagnosis and feature extraction, depicted in green. Cluster 4 consists of terms such as “cancer”, “health care”, “big data”, and “electronic health records”, representing research on the relevance of AI to cancer and chronic disease nursing in the context of medical big data, depicted in yellow. Clusters 5 and 6 have fewer nodes, including terms like “model”, “risk prediction”, and “explainable AI”, depicted in purple and light blue, respectively. Details can be seen in Figure 3A. Analysis of the appearance time of different nodes reveals the research structure and development trends of the relevant field. Figure 3B shows that almost all nodes appeared after 2020, indicating a trend of rapid development in recent years. Density analysis of the research content represented by each keyword in Figure 3C reveals that “artificial intelligence”, “machine learning”, “cancer”, and “diagnosis” are positioned at the center of the related research, indicating the highest research activity.

## Discussion

### Quantity and distribution characteristics of AI-related research in chronic disease nursing

With the rise of AI technology and the increasing prevalence of chronic diseases, research in the combined field has experienced an explosion in the past 5 years. Geographically, the largest number of publications originates from the United States, significantly surpassing other countries. Additionally, the institution with the highest publication volume is Harvard Med Sch, also from the United States. Research findings indicate that the United States began relevant research in this field as early as 2002, while China, ranked second in publication volume, began in 2012. This suggests that the United States has an early advantage in research in this field, but China's development speed is noteworthy. The emergence of Deepseek may precisely illustrate this point. Analysis of author and institution publication volume rankings reveals that research in this field is relatively dispersed, with small differences between them, indicating a need for further strengthening of cooperation to achieve mutual progress. From the analysis of cited journals, it is evident that the relevant research references journals from both computer science and electronic technology as well as medical nursing. The detection of journal bursts demonstrates the rapid development of this field. Furthermore, journals with simple and fast publication processes such as *ARXIV* are increasingly favored by scholars to cope with the rapidly changing research landscape. The overlay analysis of journal pairs reflects the mutual influence between fields. The results indicate that there is mutual citation and influence between the medical and nursing fields and the fields of computer science and mathematics.

### Current research status of AI in chronic disease nursing

Citation analysis provides an intuitive display of the main knowledge structure and trends in the field. From the results of citation clustering analysis, we can observe that the current applications of AI in chronic disease nursing mainly manifest in the following areas: (1) Screening of diabetic retinopathy. (2) AI-based medical item Internet of Things architecture. (3) Monitoring of cardiovascular diseases. (4) Detection and care of skin cancer. (5) Text mining of biomedical literature. (6) Development of artificial intelligence algorithms. (7) Detection of brain tumors. (8) Development of deep learning-based intelligent IoT health systems.

AI has demonstrated increasing potential in early disease detection through medical image analysis, with diabetic retinopathy screening being a typical example. On one hand, clinical nurses are shouldering increasingly more responsibilities, and studies have shown that transferring the task of intraocular injections to trained nurses does not increase the risk to visual function, significantly alleviating the burden of ophthalmic treatment (28, 29). On the other hand, a plethora of developed models have exhibited accuracy comparable to that of experts (30–32). The combination of these factors has led to significant advancements in the diagnosis and care of diabetic retinopathy in terms of quality and efficiency. As the incidence of skin cancer rises, more accurate assessment of suspicious skin lesions in primary care settings can reduce referrals and unnecessary biopsies, thereby improving patient experience and reducing healthcare costs. Furthermore, early diagnosis of skin cancer can improve patient prognosis. Given the crucial role of visual assessment in the diagnosis of skin diseases, AI applications can facilitate image sharing and remote healthcare and nursing (33–35). Moreover, AI has shown particularly notable performance in chronic heart disease monitoring and brain tumor research (36, 37). Besides its image analysis capabilities, AI also excels in areas such as speech recognition and natural language processing (38).

With advancements in technology, AI algorithms are experiencing continuous updates and refinements. There is a growing body of research dedicated to optimizing these algorithms to achieve optimal performance and meet diverse clinical needs. This trend further drives the development of chronic disease healthcare and nursing. As understanding of AI deepens, a systematic network is gradually forming beyond its application in specific chronic diseases, segmented into different modules. The emergence of Micro-Electro-Mechanical Systems (MEMS) has led to the development of sensors that are compact and efficient. The miniaturization of sensors has made the production of wearable sensors possible, which can collect a vast amount of data and contribute to improving individual health. Furthermore, integrating the use of wearable and miniaturized sensors with ICT technologies such as 5G/6G communication, big data, artificial intelligence, and medical Internet of Things (IoT) will enable improvements in healthcare services, personal hygiene, immunity enhancement, mental health care, and contact tracing (39).

### Analysis of research hotspots and future development trends

Based on keyword network analysis, we can summarize research hotspots and make predictions about future trends. From the analysis of research hotspots, “machine learning” and “artificial intelligence” are the most frequently occurring keywords. Additionally, high-frequency research hotspots include “breast cancer” and “computer-aided diagnosis”, indicating that these topics are currently the focus of research in the field. The results of research density analysis also confirm this observation. Furthermore, trend analysis of the appearance of hotspots reveals that research in this field is transitioning from auxiliary diagnosis and prognosis prediction of individual diseases towards the development of systematic network architectures that integrate sensors, big data, communication technologies, AI, and health care (40–42). The focus of the research shifted from “telemedicine” to “digital health”, it means that the healthcare model is shifting towards integration and personalization (43). The directions that still need further research in this field include the following aspects: (1) The widespread application of AI in chronic disease care requires a balance between technological innovation and ethical risks (44); (2) The multi system involvement characteristics of chronic diseases require deep integration of AI technology with fields such as medicine, engineering, and social sciences; (3) Enhance data standardization and interoperability, as well as improve model generalization ability (45). It is believed that the application of AI in chronic disease nursing will have a significant and far-reaching impact on future chronic disease nursing models.

### Research limitations and drawbacks

This study systematically analyzes the interdisciplinary research content at the intersection of AI and chronic disease nursing, providing insights into the development trajectory of this field. However, the study has the following limitations: (1) Due to formatting requirements of Citespace, this study only analyzes literature retrieved from the WoSCC. Other databases such as PubMed and Scopus were not included in the analysis. This may introduce a bias in the representation of research in the field, as certain relevant literature from other databases may have been overlooked. (2) The specialized terminology associated with chronic diseases and AI is highly complex, making it challenging to include all relevant terms in the search strategy. While efforts have been made to capture the research landscape, the complexity of terminology may have led to the omission of certain key terms, potentially impacting the comprehensiveness of the study's findings.

## Conclusion

To sum up, this study is the first to systematically analyze the studies on the interdisciplinary research content at the intersection of AI and chronic disease nursing using bibliometrics, and successfully establish a knowledge map in this field. The number of publications and citations related to AI research in the field of chronic disease nursing has been rapidly growing. Although AI has been paid more and more attention to the field of chronic disease nursing, there is still a lack of authors who have been engaged in this field for a long time. This bibliometric analysis not only provided a comprehensive overview to help researchers to understand the important articles, journals, potential collaborators, and institutions in this field but also analyzed the hot spots and future trends of the research topic to provide inspiration for researchers to choose research directions.

## Data Availability

The raw data supporting the conclusions of this article will be made available by the authors, without undue reservation.
